# A Patients' Perspective Towards the Injection Devices for Humira® and Imraldi® in a Nationwide Switching Program

**DOI:** 10.3389/fmed.2022.799494

**Published:** 2022-01-27

**Authors:** Kristin Karlsdottir, Anna I. Gunnarsdottir, Gerdur Grondal, Thorvardur J. Love, Elinborg Stefansdottir, Loa G. Davidsdottir, Ragna H. Thorleifsdottir, Bjorn Gudbjornsson

**Affiliations:** ^1^Hospital Pharmacy, Landspitali University Hospital, Reykjavik, Iceland; ^2^School of Health Sciences, University of Iceland, Reykjavik, Iceland; ^3^Centre for Rheumatology Research, Landspitali University Hospital, Reykjavik, Iceland; ^4^Department of Rheumatology, Landspitali University Hospital, Reykjavik, Iceland; ^5^Science and Research, Landspitali University Hospital, Reykjavik, Iceland; ^6^Department of Gastroenterology, Landspitali University Hospital, Reykjavik, Iceland; ^7^The Dermatology Center, Reykjavik, Iceland

**Keywords:** adalimumab, Humira, Imraldi, injection devices, medicine administration at home

## Abstract

**Objective:**

Due to a tender process in Iceland, all patients on Humira® were switched nationwide to its biosimilar Imraldi® in March 2019. The study aimed to explore the patient's perspective of the Humira® and Imraldi® injection devices.

**Methods:**

A standard telephone interview was carried out among patients with inflammatory arthritis, inflammatory bowel disease and psoriasis, who underwent this nationwide switching program a few months earlier.

**Results:**

The response rate was 84.5% (*n* = 198). The average age was 50.8 years, and 53.5% were female. The patients self-administered the drugs in 96% of the cases. The majority (90.5%) stated that they received individualized instruction on using the Humira® pen, compared to 18.2% who accepted instruction in the case of the Imraldi® pen. Almost half (46.6%) of the patients found it more difficult to use the Imraldi® pen than the Humira® pen, while only 12.5% found the Imraldi® pen easier to use. Firstly, these differences were due to more painful insertion of the needle (62.2%) and secondly, due to the experience, the injection process was different (63.0%).

**Conclusion:**

Patients with inflammatory disorders who have been treated regularly with adalimumab preferred the Humira® injection device over the Imraldi® device, according to our results. After all, these injection devices' structure and content are not the same, although both contain the same active ingredient, i.e. adalimumab. Our results highlight the importance of thorough information, not only with an information letter but also with the possibilities for individualized introduction in planning switching to biosimilars.

## Introduction

Since the first tumor necrosis factor-α inhibitor (TNFi) marketing in 1998, the prognosis of autoimmune diseases has changed dramatically (1). Biologic treatment is expensive, however, with the introduction of biosimilars, the cost has decreased (2). Due to the complexity of biological drugs, it is not possible to produce an accurate simulation (3, 4). Imitations of biologic drugs have the exact mechanism of action, route of administration, dosage form and concentration as the original product and must pass the same trials as the original product for safety, purity and efficacy (4). Therefore, switching from an original biologic drug to a biosimilar is considered a low-risk change (3).

The TNFi Humira® is approved for the treatment of various autoimmune diseases. In 2018 the Humira® patent expired in Europe, and its biosimilar Imraldi® was launched. Although Imraldi® has been shown to have comparable quality, efficacy and safety as Humira® (5, 6), there is a difference among the two devices used to self-administration of the drugs. Both Humira®and Imraldi® are supplied with pre-filled pens containing 40 mg of the active substance adalimumab, but Humira® contains 0.4 mL of a sterile solution while Imraldi® contains 0.8 mL. Table 1 shows the comparison between the two pens (7, 8).

**Table 1:** The table illustrate differences between two devices that contain adalimumab: Humira® pen and Imraldi® pen.

	**Humira^®^ pen**	**Imraldi^®^ pen**
**Active ingredient**	Adalimumab	Adalimumab
**Sterile solution**	0.4 mL	0.8 mL
**How to inject the drug**	Plum activator button	Button free
**Excipients**	Without citrate	With citrate
**Thickness of needle**	$\frac{1}{2}$ inch long	$\frac{1}{2}$ inch long
**Length of needle**	29 gauge	29 gauge
**Weight of the device**	35.33 g	45.87 g
**How to use the pens**	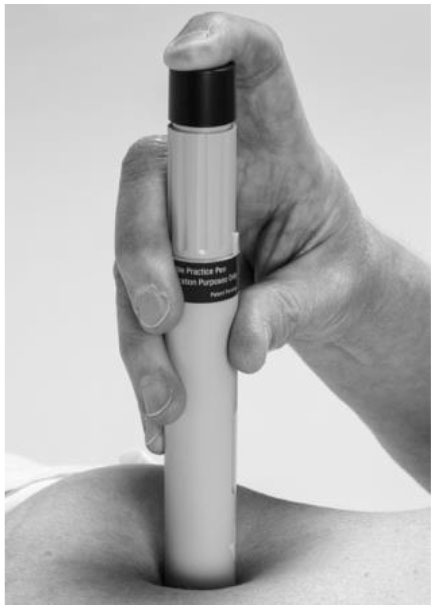	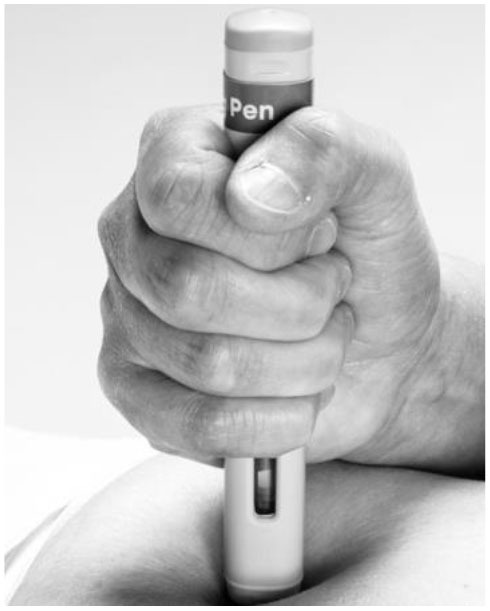

One of the excipients in Imraldi® is citrate. Some patients do not tolerate citrate as they experience discomfort when the solution is injected subcutaneously (9, 10). The original Humira® pen contained citrate, which due to this discomfort, was removed in 2018. There are also other differences in the use of these two pens. In the case of Humira®, the patient has to hold the pen stable and push a button with the thumb or index finger for the needle to come down and start the injection. In the case of Imraldi®, there is no button to push, but the patient has to hold the pen stable and firmly press it down to start the injection (7, 8) (Table 1).

Even though there has been considerable discussion about the interchangeability between biologics and biosimilars (11), there is scarce information on the patient's attitude toward such switching, especially studies comparing the injection devices. To date, no other study has been carried out to our knowledge on the transition from Humira® to Imraldi® injection devices.

## Methods

### Study Population

A quantitative study included all patients, 18-years and older, with the diagnosis of inflammatory arthritis (including rheumatoid arthritis, psoriatic arthritis and ankylosing spondylitis), inflammatory bowel disease or psoriasis treated with adalimumab by the originator Humira® and was mandatory switched to its biosimilar Imraldi® in a nationwide switching program in early spring 2019. The study was conducted from November 2019 to March 2020.

### Procedures

Due to the healthcare reimbursement system in Iceland, treating physicians need to apply annually for a license to use TNFi to the Medicine Committee at Landspitali, the University Hospital of Iceland (12). List of all patients who switched from Humira® treatment to Imraldi® from March 1^st^ 2019 and onwards due to an agreement on drug procurement was obtained from the Medicine Committee.

Information regarding diagnosis, telephone numbers and addresses were obtained from medical records. Patients who were not diagnosed with inflammatory arthritis, inflammatory bowel disease or psoriasis were excluded, as were those under 18-years. Included patients had been treated with Imraldi® for at least 3 months and were therefore experienced using the Imraldi® device and compared to their previous treatment with the Humira® device.

A questionnaire was designed to explore the patient's comparison of Humira® and Imraldi® injection devices regarding the grip, insertion of the needle and the injection from the device. The questionnaire also included questions on whether participants received instruction on using the device and from whom. The questionnaire regarding the use of these pens consisted of 7 standardized questions and one open question (Supplementary File I).

An introductory letter was sent by regular mail to all patients. Approximately 1 week later, the first author (KK) made a telephone call to the patients. After receiving oral consent for participation, a structured interview was performed according to the protocol. If the patient wanted to participate, but the time was inconvenient when they were called, a suitable time for the interview was set.

### Data Processing

The questionnaire was set up in REDCap 9.5.10 web application (Research Electronic Data Capture: Vanderbilt University, USA) and used for data collection. Microsoft Excel 2016 was used for data collection and management and graphical presentation. RStudio 1.2.5033 was used for statistical processing.

### Statistical Analysis

Descriptive analysis was used to describe the properties of the data. A chi-square and fisher's exact tests were used to evaluate statistics significance between the groups.

The study protocol was approved by the Icelandic National Bioethics Committee and Data Protective Authority in Iceland (VSNb20190100017/03.01).

## Results

Of the 234 possible participants, 198 agreed to participate, giving a response rate of 84.5%. The proportion of females was different between disease groups, with the highest among patients with inflammatory bowel disease (61.1%) and lowest in the psoriasis group (39.3%). Mean age was highest among patients with inflammatory arthritis (52.2 years ± 13.5) but lowest (46.8 years ± 14.7) in the inflammatory bowel disease group. The majority (64.6%) of participants were employed. Of those unemployed, the main reason, or in 44.3% of cases, was disability due to their underlying disease. A vast majority of participants with arthritis, or 78.4%, compared to 50.0% of the psoriasis patients and 38.9% of patients in the intestinal inflammatory disease group had previous experience with other biologic treatment before initiating treatment with Humira® (Table 2).

**Table 2:** Demographics of the 198 patients with inflammatory arthritis, inflammatory bowel disease or psoriasis who underwent mandatory switched from Humira® to Imraldi® on March 1st, 2019 due to national tender process and an agreement on drug procurement.

	**Number of** **participants with** **inflammatory** **arthritis (%)**	**Number of** **participants with** **inflammatory bowel** **disease (%)**	**Number of** **participants** **with psoriasis (%)**	**Total (%)**
**Female, number (%)**	62 (53.4)	33 (61.1)	11 (39.3)	106 (53.5)
**Mean age, years** **±SD**	52.5 ± 13.5	46.8 ± 14.7	51.5 ± 14.4	50.8 ± 14.2
**Employed (%)**	72 (62)	35 (64.8)	21 (75)	128 (64.6)
**Patients bionaive prior to treatment with Humira**^®^ **(%)**	25 (21.6)	33 (61.1)	14 (50)	72 (36.4)

### Instruction on How to Use the Humira® and Imraldi® Injection Device

Almost all patients (96%) self-administered their adalimumab injections; 99.1% of the arthritis patients, 88.9% of the inflammatory bowel disease patients and 96.4% of the psoriasis patients. Those eight patients (one arthritis patient, six inflammatory bowel patients and one psoriasis patient) who did not manage their self-administration received assistance from their spouse or other relatives.

**Figure 1:**
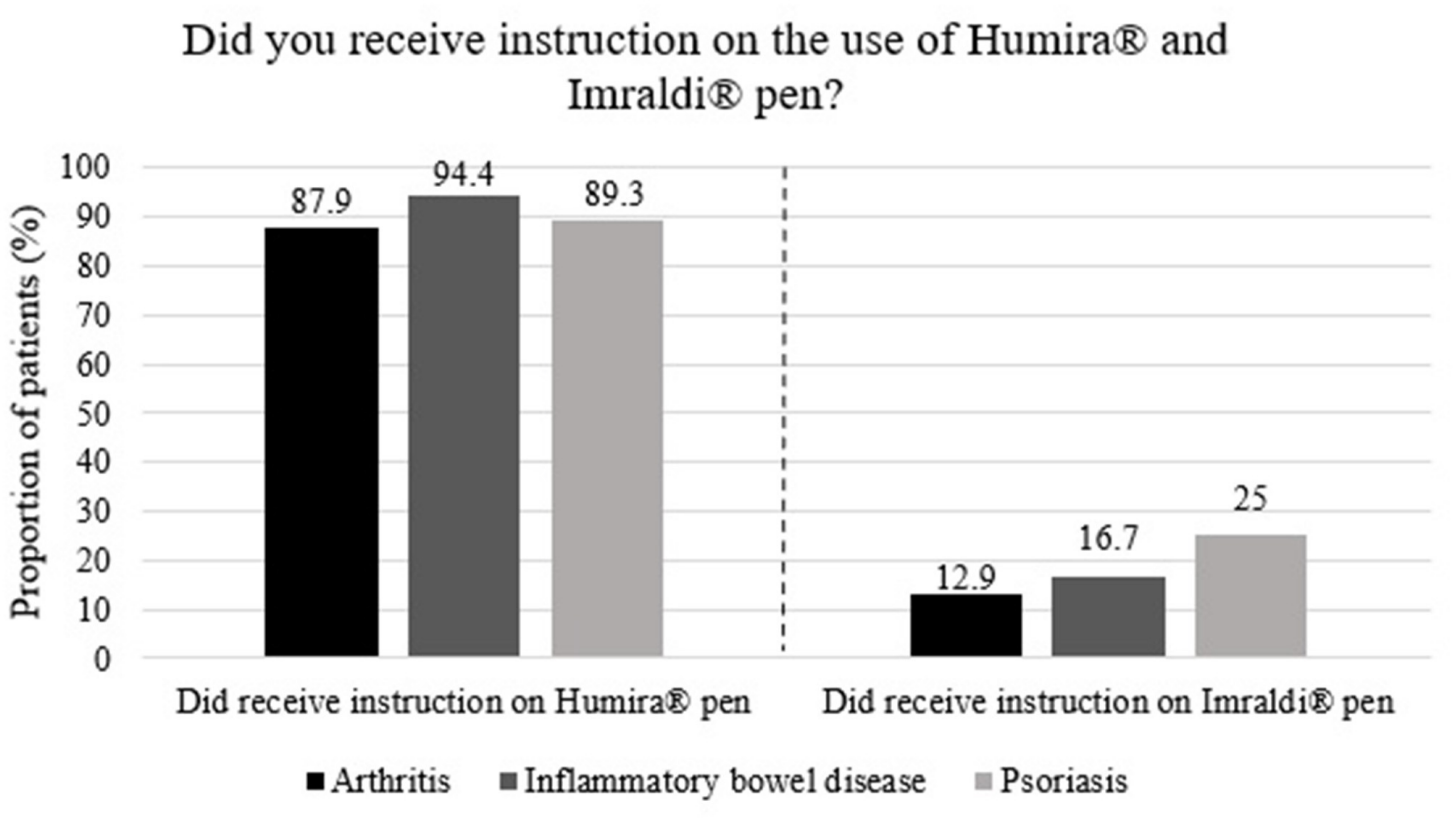
Proportion of 198 patients who received instructions on Humira® and Imraldi® injection devices.

When comparing whether patients received instructions on using the injection device, 90.5% of patients stated that they received individual introduction when the treatment with Humira® started, compared to 18.2% of patients when treatment with Imraldi® started (Figure 1). However, they had all received an information letter from Landspítali's Medicine Committee preparing for this medication switch from Humira® to Imraldi® and were encouraged to contact their doctor or nurse if further information was needed. Instruction on both pens was most commonly given at the outpatient clinic for rheumatology, gastroenterology and dermatology at Landspitali University Hospital, or in 84.4% of Humira® and 68% of Imraldi® cases.

### Comparison of the Use of the Humira® and Imraldi® Injection Devices

**Figure 2:**
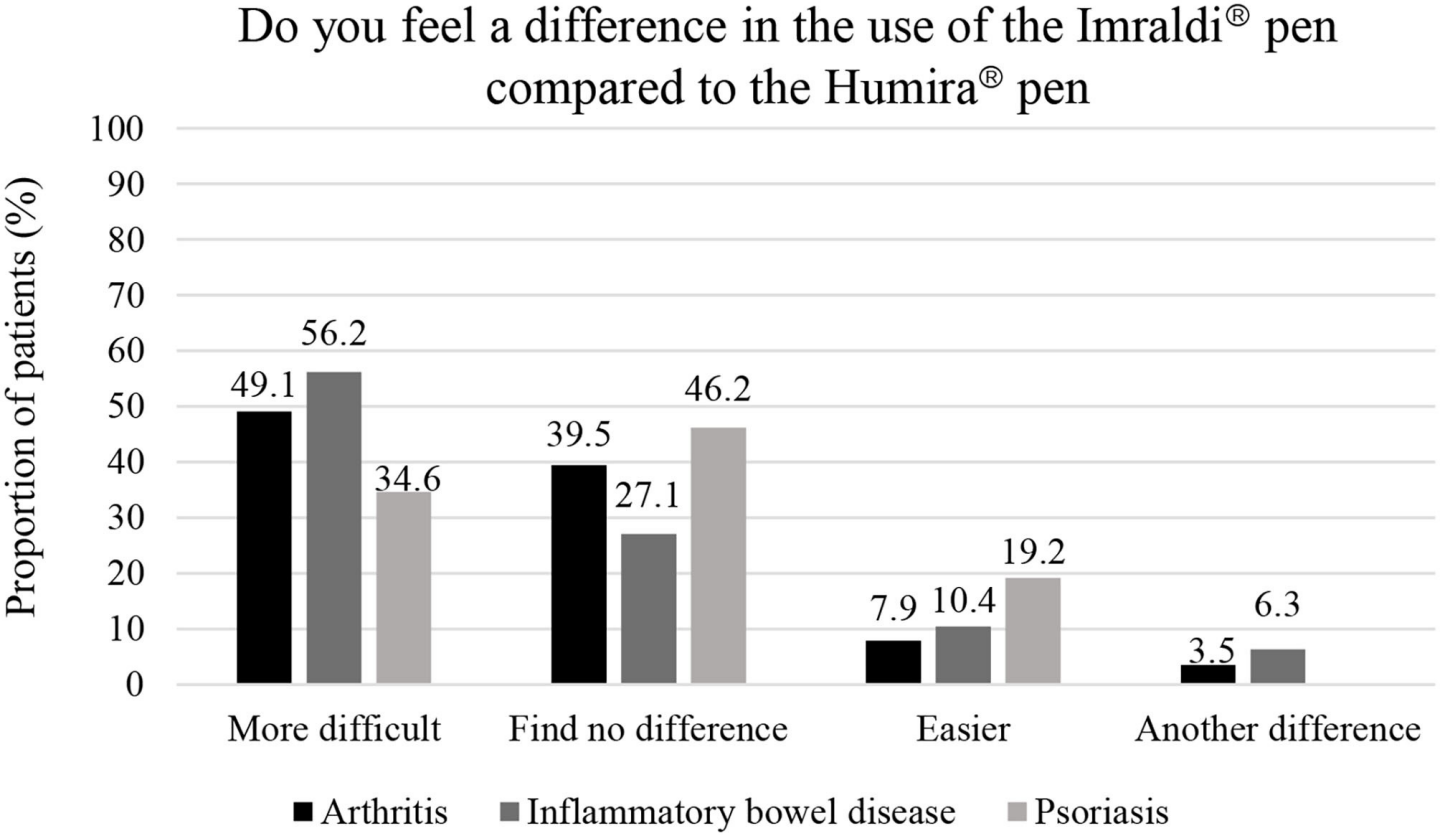
Patients' comparison of the use of Humira® and Imraldi® pens.

The majority of the patients, or 62.0%, found no difference in the grip of the two pens (Table 3). A significant difference was present in the options between finding the grip of the Imraldi® pen easier compared to finding it more difficult to use (*p* = 0.02). Additionally, a significant difference was also present in the relationship between those who found the grip of the Imraldi® pen to be more difficult and those who found no difference (*p* = 0.007). Almost half of the patients, or 46.6%, found it more challenging to use the Imraldi® pen than the Humira® pen, while 37.6% found no difference and 12.5% found the Imraldi® pen easier in use (Figure 2). No significant difference was between patients that answered more challenging or easier to use the Imraldi® pen (*p* > 0.05). Too coarse needle, painful insertion of the needle and bleeding at the injection site after administration were mentioned as the main disadvantages of the Imraldi® pen compared to the Humira® pen.

**Table 3:** Patients' comparison of the differences of the grip between the Imraldi® and the Humira® pens.

**Question: How do you find the grip of the Imraldi**^**®**^ **pen compared to the Humira**^**®**^ **pen**				
	**Arthritis** **patients** ***n*** **(%)**	**Inflammatory bowel** **diseases patients** ***n*** **(%)**	**Psoriasis patients** ***n*** **(%)**	**Total** ***n*** **(%)**
**Easier**	7 (6.2)	9 (18.8)	7 (26.9)	23 (12.3)
**More difficult**	29 (25.7)	9 (18.8)	6 (23.1)	44 (23.5)
**Another difference**	3 (2.6)	1 (2)	-	4 (2.1)
**Find no difference**	74 (65.5)	29 (60.4)	13 (50)	116 (62)

**Figure 3:**
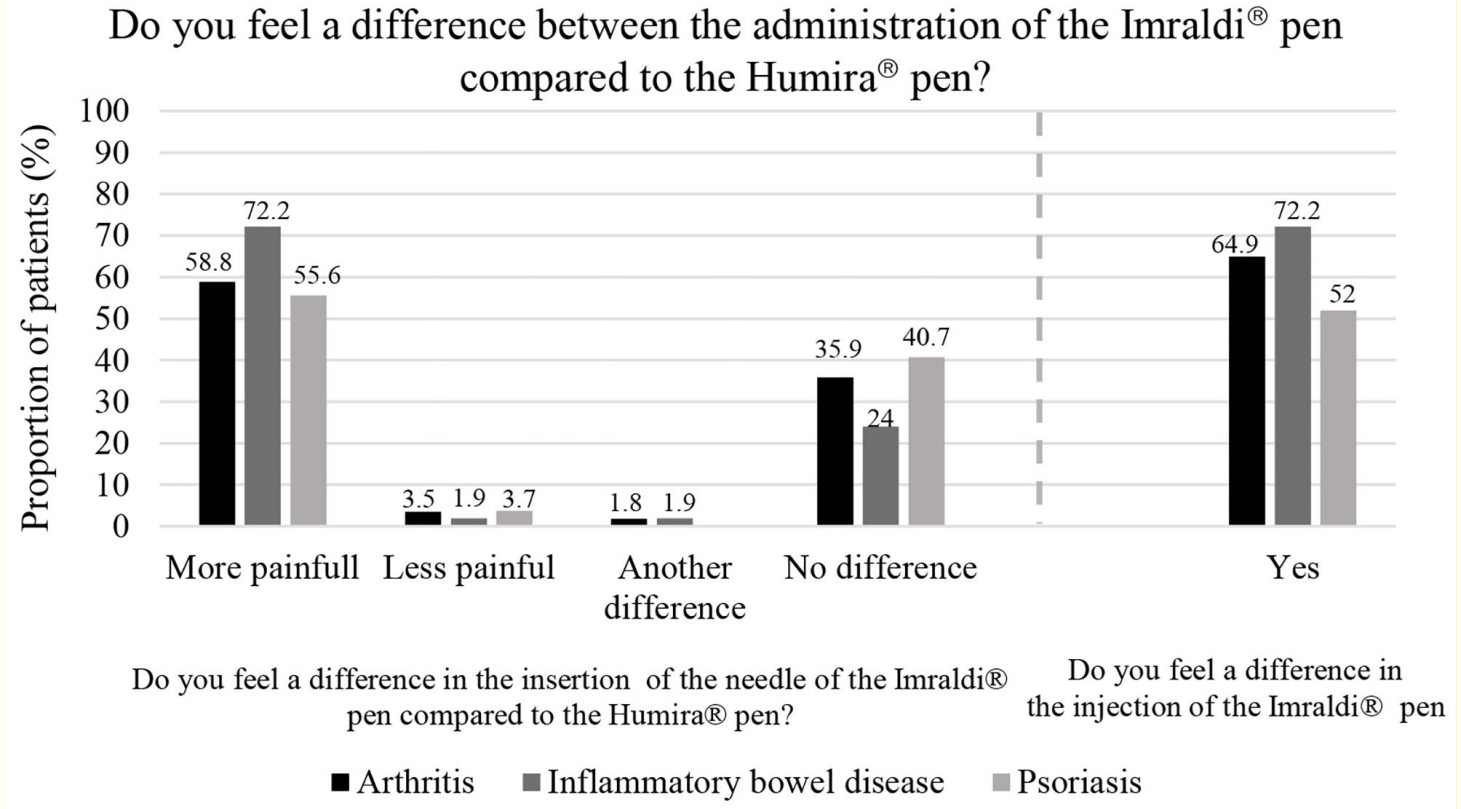
Participants' comparison of the administration of Humira® and Imraldi® pens.

Figure 3, on the left, shows that the majority of participants, or 62.2%, felt that the insertion of the needle of the Imraldi® pen was more painful compared to the Humria® pen, while very few, or 3.0%, participants felt the other way around. No significant difference was between patients that answered that the needle was more painful and the patients that thought that the needle was less painful (>0.05). Figure 3, on the right, shows that the majority of participants, 63.0%, found a difference in the injection with the Imraldi® pen compared with the Humira® pen. The most commonly mentioned difference was a more painful injection (36.8%), burning sensation during injection (28.9%) and experiencing the injection taking a more prolonged time (12.3%). However, few patients (*n* = 8 or 4%) noted a quicker injection and more shallow insertion of the needle in favor of the Imraldi® pen.

## Discussion

In the present nationwide study, we were able to run a standardized telephone interview with all patients diagnosed with inflammatory joint diseases, inflammatory bowel disorder, or psoriasis, who had been treated with the original adalimumab Humira® but were switched to its biosimilar Imraldi® due to a mandatory process in the early spring 2019. Our main findings were that majority of our patients preferred to use the Humira® pen.

When collecting data from our patients, we decided to rely on a standardized telephone interview rather than sending an e-mail or regular mail to increase the response rate and minimize missing data. The interviews were successful, and the patients were generally positive and enthusiastic about their participation. A possible reason for their willingness to participate in the study may have been their needs to express their opinion on this drug replacement. This resulted in an unprecedented participation rate of almost 85%, which secures our results' reliability.

Almost all of our patients self-administered their drug at home. It is probably based on excellent information and a person-to-person introduction on the use of the devices by nurses in outpatient specialist clinics. However, some differences in this information process might have influenced the patients' preferences for these two different pens, i.e. Humira® vs. Imraldi® pens. Almost all patients or more than 90% stated that they received individual instruction by a nurse on using the Humira® pen at the start of therapy. Although an introductory letter from the authorities was sent to all patients in this switching process where the patients were encouraged to contact their doctor or nurses for further information and the possibilities to receive a personalized introduction on the use of this new device, i.e. the Imraldi® pen, less than one fifth accepted that offer. This observation urge healthcare professionals in future switching programs to re-evaluate the information and service given in this context.

Two-thirds of our patients did not feel any differences in the two pens' grip by handling those, but a quarter of the patient thought that the hold of the Imraldi® pen was more difficult, while one-tenth thought the other way around. In addition, another two-thirds of the patients reported that they found a difference when they injected themselves with the Imraldi® pen compared with the Humira® pen. This was mainly based on the fact that the patients felt that the needle insertion by the Imraldi® pen was more painful, and they experienced a burning sensation during and after the injection. Few patients reported that the pain caused them great anxiety, and they even deliberately prolonged the time between administration. This observation is fundamental concerning the efficacy of biosimilar treatment, i.e. adherence to therapy. This could be explained by the fact that the Imraldi® solution contains more volume, and one of the excipients is citrate (7, 8). In 2018 citrate was removed from the Humira® pen since it caused pain and burning sensation (13). In fact, some of the patients mentioned that they perceived that they had returned to the “old” Humira® pen when they experienced this painful injection.

To our knowledge, no comparable study has been conducted on a nationwide basis. A survey conducted among a selected group of patients and nurses in Germany and the United Kingdom focused on the patients' preference for using Imraldi® pen versus Humira® or Enbrel® (etanercept) pens (5). Fenwick and colleagues demonstrated that most of their patients (78%) preferred to use the Imraldi® pen. Their patients considered it more comfortable to hold the Imraldi® device than the other two devices, mainly because the administration was superior, and also, the grip of the Imraldi® pen was more comfortable. Their patients also reported that they liked to have the pen “button-free” as the Imraldi® pen is. Most of the nurses who participated in the study indicated that they would recommend the Imraldi® pen to treat their patients further. In this context, we must bear in mind that this study was funded by Biogen International GmbH, the Imraldi® pen's manufacturers. Furthermore, and importantly, Fenwick used training devices without needles or active ingredients. Therefore, the patients could not comment on the difference in the needle's insertion or the injection process itself. In our study, all patients used these devices in real life, and they all had experience with the Humira® pen before switching to the Imraldi® pen. They had several months of experience with the “new” pen before participating in the present study while and our study, was independent of pharmaceutical manufacturers.

Patient experience is one of the critical parts of clinical quality, together with effectiveness and safety. Although we did not evaluate the treatment's medical efficacy on the underlying disease, we focused on patient satisfaction with the drug administration given in a medical device, i.e., prefilled pen. Our study's high response rate is also one of the main strengths of the present study, which provides essential pieces of patient experience when they undergo a mandatory drug switching between biologics and their biosimilars. Our lesson from our research has to be taken into account when planning such scenarios in the future.

The main limitations are that there are no validated standardized questionnaires concerning the aim of this study. Therefore, we designed a simple questionnaire after a thorough search and review of existing data and based on our clinical experience. However, our findings in the present study are based on individuals' opinion and experience with the various inflammatory diseases who switched from Humira® to Imraldi® and therefore did not necessarily reflect all patients who switch from any specific biologic treatment with other biologic drugs to its biosimilar. How nocebo effect may have effect our results, as these patients were mandatorily switched from Humra® to Imraldi®, is unknown. Furthermore, a randomized control trial with crossover design among biological naïve patients in respect to different devices used to self-administrate biologics would be of interest in this field of research.

In mandatory switching programs from initial biologic treatment to their biosimilars, although randomized control studies confirm the efficacy and safety of the substitution substances, the medical devices used to administer these drugs are not entirely the same. Therefore, in the early phase of all switching programs in this field, it is essential to involve individual patients and healthcare staff.

## Data Availability Statement

The original contributions presented in the study are included in the article/Supplementary Material, further inquiries can be directed to the corresponding author.

## Author Contributions

KK, AG, TL, and BG: substantial contributions to study conception and design. KK and AG: substantial contributions to acquisition of data. KK, AG, and BG: substantial contributions to analysis and interpretation of data. All authors drafting the article or revising it critically for important intellectual content and final approval of the version of the article to be published.

## Conflict of Interest

The authors declare that the research was conducted in the absence of any commercial or financial relationships that could be construed as a potential conflict of interest.

## Publisher's Note

All claims expressed in this article are solely those of the authors and do not necessarily represent those of their affiliated organizations, or those of the publisher, the editors and the reviewers. Any product that may be evaluated in this article, or claim that may be made by its manufacturer, is not guaranteed or endorsed by the publisher.
