# Supplementary material for: A Patients' Perspective Towards the Injection Devices for Humira® and Imraldi® in a Nationwide Switching Program
Source: Front Med (Lausanne). 2022 Jan 27;9:799494. doi: 10.3389/fmed.2022.799494 (PMC8829031; doi:10.3389/fmed.2022.799494)
Supplement: Supplementary file 1 [file Data_Sheet_1.PDF]

Supplementary material

Standardized telephone interview

1. **Did you receive instructions on how to use the Humira® pen before treatment started?**
    a. Yes
        i. Where did you receive the instructions?
        ii. Who gave you the instructions?
    b. No

2. **Did you receive instructions on the Imraldi® pen before treatment started?**
    a. Yes
        i. Where did you receive the instructions?
        ii. Who gave you the instructions?
    b. No

3. **Do you inject Imraldi® by yourself?**
    a. Yes
    b. No
        i. Who gives you the injection?

4. **Did you find any difference in the use of the Imraldi® pen compared to the use of the Humira® pen?**
    a. Easier
    b. Harder
    c. No difference
    d. Other difference
        i. What was different?

5. **How do you like the grip of the Imraldi® pen compared to the grip of the Humira® pen?**
    a. Easier
    b. Harder
    c. No difference
    d. Other difference
        i. What is different?

6. **Do you find any difference in the needles between Imraldi® and Humira®?**
    a. More painful
    b. Less painful
    c. No difference
    d. Other difference
        i. What is different?

7. **Do you find any difference in the injection between Imraldi® and Humira®?**
    a. Yes
        i. What is different?
    b. No

8. **Do you have any other comments conserning switching from Humira® to Imraldi®?**
    a. Open text box:
